# *N*-(3,5-Dichloro­phen­yl)-2-nitro­benzene­sulfonamide

**DOI:** 10.1107/S1600536812048283

**Published:** 2012-11-30

**Authors:** U. Chaithanya, Sabine Foro, B. Thimme Gowda

**Affiliations:** a Department of Chemistry, Mangalore University, Mangalagangotri 574 199, Mangalore, India; b Institute of Materials Science, Darmstadt University of Technology, Petersenstrasse 23, D-64287 Darmstadt, Germany

## Abstract

In the title compound, C_12_H_8_Cl_2_N_2_O_4_S, the C—S—N—C torsion angle is 49.34 (18)° and the dihedral angle between the benzene rings is 71.92 (10)°. The amide H atom exhibits bifurcated hydrogen bonding. The N—H bond is *syn* to the *ortho*-nitro group enabling the formation of an *S*(7) loop. In the crystal, pairs of N—H⋯O(S) hydrogen bonds link the mol­ecules into inversion dimers *via R*_2_^2^(8) rings.

## Related literature

For studies on the effects of substituents on the structures and other aspects of *N*-(ar­yl)-amides, see: Gowda & Weiss (1994 ▶); Shahwar *et al.* (2012 ▶), of *N*-aryl­sulfonamides, see: Chaithanya *et al.* (2012 ▶) and of *N*-chloro­aryl­sulfonamides, see: Shetty & Gowda (2004 ▶). For hydrogen-bonding patterns and motifs, see: Adsmond *et al.* (2001 ▶).
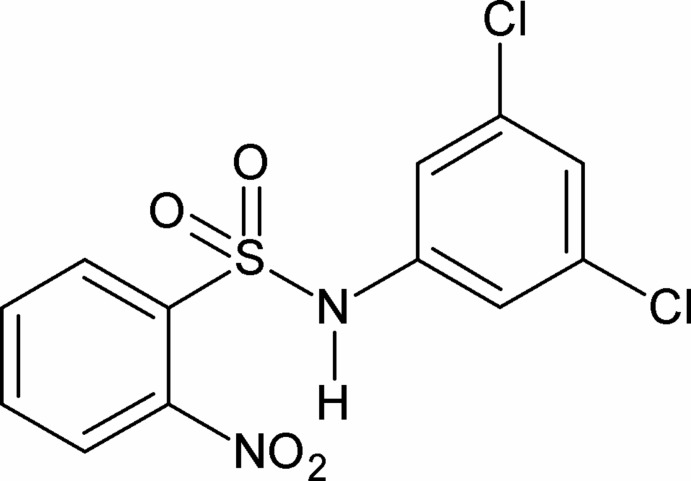


## Experimental

### 

#### Crystal data

C_12_H_8_Cl_2_N_2_O_4_S
*M* *_r_* = 347.16
Triclinic, 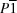

*a* = 8.2823 (8) Å
*b* = 8.3436 (9) Å
*c* = 10.670 (1) Å
α = 76.730 (8)°
β = 89.298 (9)°
γ = 86.875 (9)°
*V* = 716.59 (12) Å^3^
*Z* = 2
Mo *K*α radiation
μ = 0.61 mm^−1^
*T* = 293 K
0.44 × 0.40 × 0.28 mm

#### Data collection

Oxford Diffraction Xcalibur diffractometer with a Sapphire CCD detector
Absorption correction: multi-scan (*CrysAlis RED*; Oxford Diffraction, 2009 ▶) *T*_min_ = 0.774, *T*_max_ = 0.847
4766 measured reflections
2925 independent reflections
2600 reflections with *I* > 2σ(*I*)
*R*_int_ = 0.009

#### Refinement

*R*[*F* ^2^ > 2σ(*F* ^2^)] = 0.035
*wR*(*F* ^2^) = 0.092
*S* = 1.04
2925 reflections
194 parameters
1 restraint
H atoms treated by a mixture of independent and constrained refinement
Δρ_max_ = 0.44 e Å^−3^
Δρ_min_ = −0.49 e Å^−3^

### 

Data collection: *CrysAlis CCD* (Oxford Diffraction, 2009 ▶); cell refinement: *CrysAlis CCD*; data reduction: *CrysAlis RED* (Oxford Diffraction, 2009 ▶); program(s) used to solve structure: *SHELXS97* (Sheldrick, 2008 ▶); program(s) used to refine structure: *SHELXL97* (Sheldrick, 2008 ▶); molecular graphics: *PLATON* (Spek, 2009 ▶); software used to prepare material for publication: *SHELXL97*.

## Supplementary Material

Crystal structure: contains datablock(s) I, global. DOI: 10.1107/S1600536812048283/tk5173sup1.cif

Structure factors: contains datablock(s) I. DOI: 10.1107/S1600536812048283/tk5173Isup2.hkl

Supplementary material file. DOI: 10.1107/S1600536812048283/tk5173Isup3.cml

Additional supplementary materials:  crystallographic information; 3D view; checkCIF report

## Figures and Tables

**Table 1:** Hydrogen-bond geometry (Å, °)

*D*—H⋯*A*	*D*—H	H⋯*A*	*D*⋯*A*	*D*—H⋯*A*
N1—H1*N*⋯O2^i^	0.85 (2)	2.23 (2)	3.052 (2)	162 (2)
N1—H1*N*⋯O3	0.85 (2)	2.44 (2)	2.940 (2)	118 (2)

Symmetry code: (i) 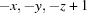.

**Fig. 1.**
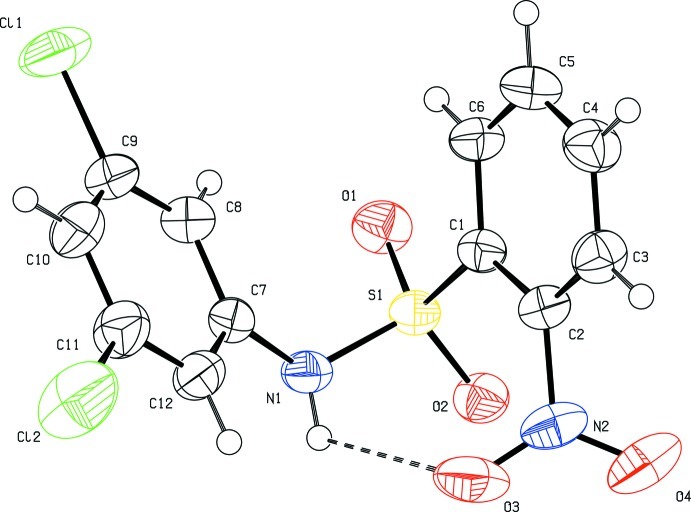
Molecular structure of the title compound, showing the atom labelling scheme and with displacement ellipsoids drawn at the 50% probability level. The dashed line indicates an intramolecular N—H···O hydrogen bond.

**Fig. 2.**
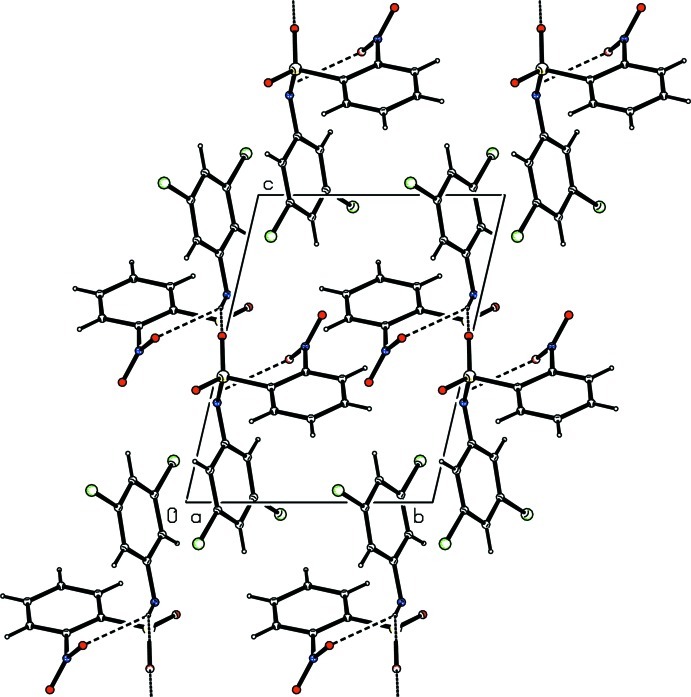
Molecular packing of the title compound with hydrogen bonding shown as dashed lines.

## supplementary crystallographic information

### Comment

As a part of our studies on the substituent effects on the structures and other aspects of *N*-(aryl)-amides (Gowda & Weiss, 1994; Shahwar *et al.*, 2012); *N*-arylsulfonamides (Chaithanya *et al.*, 2012); and *N*-chloroarylsulfonamides (Shetty & Gowda, 2004), in the present work, the crystal structure of *N*-(3,5-dichlorophenyl)-2-nitrobenzenesulfonamide (I) has been determined (Fig. 1).

The conformation of the N—C bond in the —SO_2_—NH—C segment has *gauche* torsions with respect to the S═O bonds (Fig. 1), similar to that observed in *N*-(3,5-dimethylphenyl)-2-nitrobenzenesulfonamide (II) (Chaithanya *et al.*, 2012). Further, the conformation of the N—H bond in the —SO_2_—NH— segment is *syn* to the *ortho*- nitro group in the sulfonyl benzene ring. The molecule is twisted at the S—N bond with the torsional angle of 49.34 (18)°, compared to the values of 44.24 (26) and -49.34 (25)° in the two independent molecules of (II).

The dihedral angle between the sulfonyl and the aniline rings in (I) is 71.92 (10)°, compared to the values of 71.53 (7)° and 72.11 (7)° in the two molecules of (II).

The amide H-atom shows bifurcated intramolecular H-bonding with the O-atom of the *ortho*-nitro group in the sulfonyl benzene ring, generating S(7) motifs, and the intermolecular H-bonding with the sulfonyl oxygen atom of a symmetry related molecule, generating *R*^2^_2_(8) motifs (Adsmond *et al.*, 2001). The latter (Table 1) link the molecules into inversion dimers. Part of the crystal structure is shown in Fig. 2.

### Experimental

The title compound was prepared by treating 2-nitrobenzenesulfonylchloride with 3,5-dichloroaniline in a stoichiometric ratio and boiling the reaction mixture for 15 minutes. The reaction mixture was then cooled to room temperature and added to ice cold water (100 ml). The resultant solid, *N*-(3,5-dichlorophenyl)-2-nitrobenzenesulfonamide, was filtered under suction and washed thoroughly with cold water and dilute HCl to remove the excess sulfonylchloride and aniline, respectively. It was then recrystallized to constant melting point from dilute ethanol.

Prism-like colourless crystals of the title compound used in X-ray diffraction studies were grown from its ethanolic solution by slow evaporation of the solvent at room temperature.

### Refinement

H atoms bonded to C were positioned with idealized geometry using a riding model with C—H = 0.93 Å. The amino H atom was refined with the N—H distance restrained to 0.86 (2) Å. All H atoms were refined with isotropic displacement parameters set at 1.2 *U*_eq_ of the parent atom.

### Crystal data

**Table:**

C_12_H_8_Cl_2_N_2_O_4_S	*Z* = 2
*M**_r_* = 347.16	*F*(000) = 352
Triclinic, *P*1	*D*_x_ = 1.609 Mg m^−^^3^
Hall symbol: -P 1	Mo *K*α radiation, λ = 0.71073 Å
*a* = 8.2823 (8) Å	Cell parameters from 3256 reflections
*b* = 8.3436 (9) Å	θ = 3.1–27.7°
*c* = 10.670 (1) Å	µ = 0.61 mm^−^^1^
α = 76.730 (8)°	*T* = 293 K
β = 89.298 (9)°	Prism, colourless
γ = 86.875 (9)°	0.44 × 0.40 × 0.28 mm
*V* = 716.59 (12) Å^3^	

### Data collection

**Table:**

Oxford Diffraction Xcalibur diffractometer with a Sapphire CCD detector	2925 independent reflections
Radiation source: fine-focus sealed tube	2600 reflections with *I* > 2σ(*I*)
Graphite monochromator	*R*_int_ = 0.009
Rotation method data acquisition using ω scans	θ_max_ = 26.4°, θ_min_ = 3.2°
Absorption correction: multi-scan (*CrysAlis RED*; Oxford Diffraction, 2009)	*h* = −8→10
*T*_min_ = 0.774, *T*_max_ = 0.847	*k* = −9→10
4766 measured reflections	*l* = −7→13

### Refinement

**Table:**

Refinement on *F*^2^	Secondary atom site location: difference Fourier map
Least-squares matrix: full	Hydrogen site location: inferred from neighbouring sites
*R*[*F*^2^ > 2σ(*F*^2^)] = 0.035	H atoms treated by a mixture of independent and constrained refinement
*wR*(*F*^2^) = 0.092	*w* = 1/[σ^2^(*F*_o_^2^) + (0.0408*P*)^2^ + 0.4244*P*] where *P* = (*F*_o_^2^ + 2*F*_c_^2^)/3
*S* = 1.04	(Δ/σ)_max_ < 0.001
2925 reflections	Δρ_max_ = 0.44 e Å^−^^3^
194 parameters	Δρ_min_ = −0.49 e Å^−^^3^
1 restraint	Extinction correction: *SHELXL97* (Sheldrick, 2008), Fc^*^=kFc[1+0.001xFc^2^λ^3^/sin(2θ)]^-1/4^
Primary atom site location: structure-invariant direct methods	Extinction coefficient: 0.100 (5)

### Special details

**Table:**

Experimental. Absorption correction: CrysAlis RED (Oxford Diffraction, 2009) Empirical absorption correction using spherical harmonics, implemented in SCALE3 ABSPACK scaling algorithm.
Geometry. All e.s.d.'s (except the e.s.d. in the dihedral angle between two l.s. planes) are estimated using the full covariance matrix. The cell e.s.d.'s are taken into account individually in the estimation of e.s.d.'s in distances, angles and torsion angles; correlations between e.s.d.'s in cell parameters are only used when they are defined by crystal symmetry. An approximate (isotropic) treatment of cell e.s.d.'s is used for estimating e.s.d.'s involving l.s. planes.
Refinement. Refinement of *F*^2^ against ALL reflections. The weighted *R*-factor *wR* and goodness of fit *S* are based on *F*^2^, conventional *R*-factors *R* are based on *F*, with *F* set to zero for negative *F*^2^. The threshold expression of *F*^2^ > σ(*F*^2^) is used only for calculating *R*-factors(gt) *etc*. and is not relevant to the choice of reflections for refinement. *R*-factors based on *F*^2^ are statistically about twice as large as those based on *F*, and *R*- factors based on ALL data will be even larger.

### Fractional atomic coordinates and isotropic or equivalent isotropic displacement parameters (Å^2^)

**Table:**

	*x*	*y*	*z*	*U*_iso_*/*U*_eq_	
C1	0.3367 (2)	0.2350 (2)	0.37443 (17)	0.0340 (4)	
C2	0.2649 (2)	0.3607 (2)	0.42524 (17)	0.0371 (4)	
C3	0.3229 (3)	0.5157 (2)	0.4011 (2)	0.0459 (5)	
H3	0.2729	0.5973	0.4369	0.055*	
C4	0.4565 (3)	0.5488 (3)	0.3228 (2)	0.0492 (5)	
H4	0.4964	0.6537	0.3046	0.059*	
C5	0.5305 (2)	0.4268 (3)	0.2718 (2)	0.0498 (5)	
H5	0.6207	0.4496	0.2192	0.060*	
C6	0.4721 (2)	0.2700 (2)	0.29780 (19)	0.0419 (4)	
H6	0.5241	0.1880	0.2636	0.050*	
C7	0.0975 (2)	0.1026 (2)	0.19048 (18)	0.0380 (4)	
C8	0.2129 (2)	0.0663 (3)	0.1044 (2)	0.0448 (4)	
H8	0.3031	−0.0032	0.1331	0.054*	
C9	0.1901 (3)	0.1361 (3)	−0.0251 (2)	0.0495 (5)	
C10	0.0575 (3)	0.2374 (3)	−0.0718 (2)	0.0560 (6)	
H10	0.0443	0.2824	−0.1594	0.067*	
C11	−0.0553 (3)	0.2695 (3)	0.0166 (2)	0.0549 (5)	
C12	−0.0370 (2)	0.2058 (3)	0.14680 (19)	0.0472 (5)	
H12	−0.1136	0.2315	0.2046	0.057*	
N1	0.10830 (19)	0.0311 (2)	0.32445 (15)	0.0403 (4)	
H1N	0.024 (2)	0.045 (3)	0.367 (2)	0.048*	
N2	0.1200 (2)	0.3345 (2)	0.50690 (17)	0.0481 (4)	
O1	0.39505 (17)	−0.06691 (17)	0.36502 (15)	0.0478 (3)	
O2	0.21976 (16)	−0.01250 (17)	0.54098 (13)	0.0438 (3)	
O3	0.00227 (19)	0.2822 (2)	0.4655 (2)	0.0675 (5)	
O4	0.1245 (3)	0.3732 (2)	0.60935 (17)	0.0764 (6)	
Cl1	0.33194 (8)	0.08990 (10)	−0.13460 (6)	0.0734 (2)	
Cl2	−0.22590 (10)	0.39424 (13)	−0.03893 (7)	0.0972 (3)	
S1	0.27077 (5)	0.03037 (5)	0.40942 (4)	0.03530 (15)	

### Atomic displacement parameters (Å^2^)

**Table:**

	*U*^11^	*U*^22^	*U*^33^	*U*^12^	*U*^13^	*U*^23^
C1	0.0303 (8)	0.0350 (9)	0.0351 (9)	−0.0007 (7)	0.0013 (7)	−0.0052 (7)
C2	0.0361 (9)	0.0384 (9)	0.0349 (9)	0.0005 (7)	0.0055 (7)	−0.0054 (7)
C3	0.0510 (11)	0.0397 (10)	0.0480 (11)	−0.0017 (8)	0.0055 (9)	−0.0121 (8)
C4	0.0502 (11)	0.0416 (10)	0.0550 (12)	−0.0117 (9)	0.0049 (9)	−0.0074 (9)
C5	0.0388 (10)	0.0546 (12)	0.0541 (12)	−0.0114 (9)	0.0127 (9)	−0.0074 (10)
C6	0.0343 (9)	0.0461 (11)	0.0452 (10)	−0.0008 (8)	0.0080 (8)	−0.0113 (8)
C7	0.0370 (9)	0.0437 (10)	0.0349 (9)	−0.0102 (8)	0.0032 (7)	−0.0104 (8)
C8	0.0386 (10)	0.0517 (11)	0.0449 (11)	−0.0060 (8)	0.0070 (8)	−0.0123 (9)
C9	0.0472 (11)	0.0633 (13)	0.0411 (11)	−0.0130 (10)	0.0143 (9)	−0.0171 (10)
C10	0.0607 (13)	0.0719 (15)	0.0347 (10)	−0.0075 (11)	0.0036 (9)	−0.0101 (10)
C11	0.0515 (12)	0.0702 (15)	0.0413 (11)	0.0051 (11)	−0.0041 (9)	−0.0105 (10)
C12	0.0415 (10)	0.0637 (13)	0.0375 (10)	0.0027 (9)	0.0026 (8)	−0.0152 (9)
N1	0.0330 (8)	0.0512 (9)	0.0356 (8)	−0.0068 (7)	0.0055 (6)	−0.0070 (7)
N2	0.0528 (10)	0.0374 (9)	0.0497 (10)	0.0056 (7)	0.0200 (8)	−0.0040 (7)
O1	0.0430 (7)	0.0395 (7)	0.0611 (9)	0.0055 (6)	0.0042 (6)	−0.0141 (6)
O2	0.0438 (7)	0.0464 (8)	0.0363 (7)	−0.0034 (6)	0.0012 (6)	0.0008 (6)
O3	0.0427 (8)	0.0656 (11)	0.0972 (14)	−0.0082 (8)	0.0266 (9)	−0.0246 (10)
O4	0.0956 (14)	0.0836 (13)	0.0489 (10)	0.0012 (11)	0.0302 (9)	−0.0156 (9)
Cl1	0.0674 (4)	0.0992 (5)	0.0540 (4)	−0.0063 (3)	0.0297 (3)	−0.0193 (3)
Cl2	0.0839 (5)	0.1405 (8)	0.0550 (4)	0.0480 (5)	−0.0145 (3)	−0.0092 (4)
S1	0.0331 (2)	0.0330 (2)	0.0379 (3)	0.00006 (16)	0.00272 (17)	−0.00480 (17)

### Geometric parameters (Å, º)

**Table:**

C1—C6	1.384 (2)	C8—C9	1.382 (3)
C1—C2	1.390 (3)	C8—H8	0.9300
C1—S1	1.7763 (18)	C9—C10	1.374 (3)
C2—C3	1.372 (3)	C9—Cl1	1.737 (2)
C2—N2	1.470 (2)	C10—C11	1.378 (3)
C3—C4	1.379 (3)	C10—H10	0.9300
C3—H3	0.9300	C11—C12	1.376 (3)
C4—C5	1.374 (3)	C11—Cl2	1.735 (2)
C4—H4	0.9300	C12—H12	0.9300
C5—C6	1.386 (3)	N1—S1	1.6308 (16)
C5—H5	0.9300	N1—H1N	0.848 (16)
C6—H6	0.9300	N2—O4	1.210 (2)
C7—C8	1.387 (3)	N2—O3	1.217 (3)
C7—C12	1.387 (3)	O1—S1	1.4200 (14)
C7—N1	1.419 (2)	O2—S1	1.4312 (14)
			
C6—C1—C2	117.76 (17)	C10—C9—C8	122.82 (19)
C6—C1—S1	118.50 (14)	C10—C9—Cl1	118.30 (17)
C2—C1—S1	123.64 (13)	C8—C9—Cl1	118.86 (18)
C3—C2—C1	122.36 (17)	C9—C10—C11	117.3 (2)
C3—C2—N2	116.28 (17)	C9—C10—H10	121.3
C1—C2—N2	121.35 (16)	C11—C10—H10	121.3
C2—C3—C4	118.96 (19)	C12—C11—C10	122.3 (2)
C2—C3—H3	120.5	C12—C11—Cl2	119.11 (18)
C4—C3—H3	120.5	C10—C11—Cl2	118.64 (18)
C5—C4—C3	119.99 (19)	C11—C12—C7	118.90 (19)
C5—C4—H4	120.0	C11—C12—H12	120.6
C3—C4—H4	120.0	C7—C12—H12	120.6
C4—C5—C6	120.64 (18)	C7—N1—S1	123.42 (13)
C4—C5—H5	119.7	C7—N1—H1N	114.9 (16)
C6—C5—H5	119.7	S1—N1—H1N	111.2 (16)
C1—C6—C5	120.27 (18)	O4—N2—O3	124.45 (19)
C1—C6—H6	119.9	O4—N2—C2	117.1 (2)
C5—C6—H6	119.9	O3—N2—C2	118.40 (18)
C8—C7—C12	120.52 (18)	O1—S1—O2	119.91 (9)
C8—C7—N1	121.79 (18)	O1—S1—N1	108.52 (9)
C12—C7—N1	117.63 (17)	O2—S1—N1	105.46 (8)
C9—C8—C7	118.2 (2)	O1—S1—C1	106.10 (8)
C9—C8—H8	120.9	O2—S1—C1	108.89 (8)
C7—C8—H8	120.9	N1—S1—C1	107.41 (8)
			
C6—C1—C2—C3	0.5 (3)	C10—C11—C12—C7	−1.5 (4)
S1—C1—C2—C3	177.01 (15)	Cl2—C11—C12—C7	178.31 (17)
C6—C1—C2—N2	179.58 (17)	C8—C7—C12—C11	1.0 (3)
S1—C1—C2—N2	−3.9 (3)	N1—C7—C12—C11	−176.4 (2)
C1—C2—C3—C4	0.5 (3)	C8—C7—N1—S1	50.4 (2)
N2—C2—C3—C4	−178.64 (19)	C12—C7—N1—S1	−132.29 (17)
C2—C3—C4—C5	−0.8 (3)	C3—C2—N2—O4	−52.3 (3)
C3—C4—C5—C6	0.2 (3)	C1—C2—N2—O4	128.6 (2)
C2—C1—C6—C5	−1.2 (3)	C3—C2—N2—O3	124.8 (2)
S1—C1—C6—C5	−177.86 (16)	C1—C2—N2—O3	−54.3 (3)
C4—C5—C6—C1	0.9 (3)	C7—N1—S1—O1	−64.96 (18)
C12—C7—C8—C9	0.2 (3)	C7—N1—S1—O2	165.37 (15)
N1—C7—C8—C9	177.49 (18)	C7—N1—S1—C1	49.34 (18)
C7—C8—C9—C10	−1.0 (3)	C6—C1—S1—O1	9.79 (17)
C7—C8—C9—Cl1	−179.14 (15)	C2—C1—S1—O1	−166.71 (16)
C8—C9—C10—C11	0.5 (4)	C6—C1—S1—O2	140.13 (15)
Cl1—C9—C10—C11	178.63 (18)	C2—C1—S1—O2	−36.37 (18)
C9—C10—C11—C12	0.8 (4)	C6—C1—S1—N1	−106.13 (16)
C9—C10—C11—Cl2	−179.01 (19)	C2—C1—S1—N1	77.38 (17)

### Hydrogen-bond geometry (Å, º)

**Table:**

*D*—H···*A*	*D*—H	H···*A*	*D*···*A*	*D*—H···*A*
N1—H1*N*···O2^i^	0.85 (2)	2.23 (2)	3.052 (2)	162 (2)
N1—H1*N*···O3	0.85 (2)	2.44 (2)	2.940 (2)	118 (2)

Symmetry code: (i) −*x*, −*y*, −*z*+1.
